# A Model Patient

**Published:** 1861-10

**Authors:** 


### A MODEL PATIENT.

Miss C—— has been a patient of ours about four years, her teeth are of rather frail texture, and in fine condition for that class of teeth. She exercises the most scrupulous care of them, and does not permit a particle of stain or deposit to remain upon them; she has ten or twelve teeth filled, all small fillings. In all the fillings made in the last two years, she first detected the cavities, though they were small; she has in three or four instances detected minute decays on the proximal surfaces of the teeth where they stood in contact, and separated them with cotton sufficient to be filled conveniently, and had them ready to fill when we first saw them. She understands perfectly the importance of attention to incipient decay, and acts promptly upon that knowledge. How glorious to be a dentist, with five hundred such patients. T.

